# Caffeine prevents hyperoxia-induced lung injury in neonatal mice through NLRP3 inflammasome and NF-κB pathway

**DOI:** 10.1186/s12931-020-01403-2

**Published:** 2020-06-08

**Authors:** Shangqin Chen, Qiuping Wu, Dingjuan Zhong, Changchong Li, Lizhong Du

**Affiliations:** 1 Department of Pediatrics, The Children’s Hospital, Zhejiang University School of Medicine, National Clinical Research Center for Child Health, Zhejiang, Hangzhou China; 2 Department of Neonatology, The Second Affiliated Hospital & Yuying Children’s Hospital of Wenzhou Medical University, Wenzhou, Zhejiang China; 3 Department of Pediatric Pulmonology, The Second Affiliated Hospital & Yuying Children’s Hospital of Wenzhou Medical University, Wenzhou, Zhejiang China; 4 Molecular Center for Ophthalmic Optics, Hunan Provincial People’s Hospital, Changsha, China; 5 Neuropharmacology Laboratory, School of Optometry and Ophthalmology and Eye Hospital, Wenzhou Medical University, Wenzhou, China; 6 Department of Neonatology, The Children’s Hospital, Zhejiang University School of Medicine, National Clinical Research Center for Child Health, No.3333 Binsheng Road, Hangzhou, 310052 Zhejiang China

**Keywords:** Bronchopulmonary dysplasia, Oxidative stress, Caffeine, Inflammasome, NF-κB pathway

## Abstract

**Background:**

Bronchopulmonary dysplasia (BPD) is a common chronic lung disease in premature infants and hyperoxia exposure is a major cause. In hyperoxic lung injury animal model, alveolar simplification and pro-inflammatory cells infiltration are the main pathophysiologic changes. Caffeine is a drug used to treat apnea in premature infants. Early use of caffeine can decrease the rate and the severity of BPD while the mechanisms are still unclear. The purpose of this study was to evaluate the effects of caffeine on inflammation and lung development in neonatal mice with hyperoxic lung injury and to explore the possible mechanism.

**Methods:**

Following 14 d of 75% oxygen exposure in newborn mouse, the BPD model was established. Caffeine at a dose of 1 g/L was added in drinking water to nursing mouse. We measured the concentration of caffeine in serum and oxidative stress in lung by commercially available kits. Adenosine 2A receptor (A_2A_R) expression and lung inflammation were measured by Immunohistochemistry and western blotting. Apoptosis and surfactant protein-C (SFTPC) levels were measured by immunofluorescence. The inflammasome and NF-κB pathway proteins were assessed by western blotting.

**Results:**

We found that the caffeine concentration in plasma at present dose significantly decreased the expression of A_2A_R protein in mice lung. Caffeine treatment significantly reduced oxidative stress, improved weight gain, promoted alveolar development, attenuated inflammatory infiltration and lung injury in hyperoxia-induced lung injury mice. Moreover, caffeine decreased the cell apoptosis in lung tissues, especially the Type II alveolar epithelial cell. The expression of NLRP3 inflammasome protein and NF-κB pathway were significantly inhibited by caffeine treatment.

**Conclusion:**

Caffeine treatment can protect hyperoxia-induced mice lung from oxidative injury by inhibiting NLRP3 inflammasome and NF-κB pathway.

## Background

Bronchopulmonary dysplasia (BPD) is a common chronic lung disease (CLD) in premature infants, especially those with extremely low birth weight or / and extremely preterm [1]. Alveolar simplification is the main BPD-associated pathological change observed in the lungs that impacts effective gas exchange and lung function [2, 3]. Oxygen toxicity is a major risk factor for premature infants to develop BPD. When premature lung is exposed to high oxygen level, excessive oxidative stress will overtake the scavenging abilities of immature organ system [4, 5]. As a result, excess reactive oxygen species (ROS) can activate specific inflammatory cells, increase the inflammatory cytokines and proteins level in the lung, resulted in lung injury and cell death, particularly alveolar epithelial cells.

The excessive production and release of inflammatory cytokines play important roles in the mechanism of hyperoxic induced acute lung injury (HALI) [6]. Many studies have shown that an increase of pro-inflammatory factors, such as IL-6, IL-1β, and TNF-α, may activate cyto-immune responses, damage lung epithelial and endothelial cells and result in the development of BPD [7–9]. Among these proinflammatory factors, cyclooxygenase (COX) enzymes are considered as important rate-limiting enzymes involved in inflammatory tissue injury. Notably, COX-2 expression is significantly increased in inflammatory disease and closely related to neutrophil, monocyte function [10, 11]. Additionally, myeloperoxidase (MPO) is an important factor which is closely related to neutrophils activation [12].

Currently, IL-1β has been confirmed to activate inflammasome formation, which is a biomarker reflecting the cellular immune stress response. Inflammasome is a multi-protein complex which mainly combine with Caspase-1 [13]. Among the proteins in the inflammasome, NLRP3 is the most widely studied and an increased level can be found during oxidative stress and systemic infections [14, 15]. NLRP3, ASC and Caspase-1 are the main components of the NLRP3 inflammasome [16]. Grailer et al. found that compared with control mice, Caspase-1 and NLRP3 knockout mice could reduce the albumin leak and IL-1β expression in BALF, which affected the numbers of neutrophils in LPS-induced ALI mice [17]. Yoshiko found that NLRP3 could affect macrophage and neutrophil function and contribute to the pathophysiology of HALI [18]. Liao found that the NLRP3 inflammasome was a key mechanism in the development of BPD [19].

Caffeine is a methylxanthine drug used to prevent and treat apnea in premature infants in the neonatal intensive care unit (NICU). As reported, Caffeine can effectively block nonspecific adenosine receptors (AR) and weaken the inhibitory effect of neurotransmitters on respiratory drive [20]. A_2A_R is a G protein-coupled receptor which had a high affinity to caffeine, and can activate a series of downstream pathways in brain injury [21]. In a clinical study, the early use of caffeine was found to reduce the incidence of BPD and the mechanical ventilation time [22]. In animal models, Teng found that caffeine reduced endoplasmic reticulum stress levels in hyperoxia-induced lung injury rats [23]. Fehrholz found caffeine had a significant anti-inflammatory effect and down-regulated the abnormal Smad pathway in inflammatory A549 cells [24].

NF-κB is a key transcription factor related to inflammation, which can stimulate inflammatory response and cause cell apoptosis and injury by regulating inflammatory related factors. When THP-1 macrophages were exposed to LPS, Zhao found that caffeine inhibited NF-κB pathway, reduced the inflammasomes activation, and decreased cell apoptosis [25]. However, the specific mechanism on oxidative stress and NF-κB pathway in hyperoxia mice following caffeine treatment remains unclear.

In our present study, we investigated the effect of caffeine on inflammation and lung injury in a mouse model of BPD. The oxidative stress, inflammatory reactions, and especially the role of the inflammasome and NF-κB pathways in the caffeine-mediated protective effects were evaluated.

## Materials and methods

### Animals and study design

C57BL/6 mice were obtained from the Animal Center of the Chinese Academy of Science (Shanghai, China). All animal experiments were conducted in compliance with the Guidelines for the Care and Use of Laboratory Animals from the National Institutes of Health and were approved by the laboratory Animal Ethics Committee of Wenzhou Medical University (the approval code number is wydw2019–0695).

Adult mice were crossed to deliver litters for subsequent studies. At full term (d 21–22 of pregnancy), the dams delivered naturally, and the pups were pooled, randomized, and returned to the nursing dams within 6 h. Afterwards, the pups were divided into four experimental groups: the Normoxia (NO) group; the Normoxia + Caffeine (NC) group; the Hyperoxia (HO) group; and the Hyperoxia + Caffeine (HC) group. A solution of 1 g/L caffeine (Sigma Aldrich, St. Louis, USA) was prepared in drinking water and administered to nursing mothers from postnatal day 0 (P0)-P14 as described previously [26]. The mice in the Normoxia and Hyperoxia groups were exposed to drinking water without caffeine. The mice exposed to normoxia were placed in room air with 21% oxygen, and the mice exposed to hyperoxia were placed in 75% oxygen for 14 days. The continuous exposure to 75% O_2_ was achieved in a Plexiglass chamber flow-through system, and the O_2_ level inside was continuously monitored with an O_2_ analyzer. Nursing dams (HO with NO group dams, HC with NC group dams) were rotated between the litters exposed to hyperoxia and normoxia every 24 h to prevent O_2_ toxicity. The chamber was opened once per day for 0.5 h to replace the food and water. The body weight of the mice in each group was determined on P7 and P14.

All neonatal mice were survived during the entire experimental period. The mice in each group were sacrificed on P14 by an intraperitoneal injection of 1% pentobarbital (50 mg/kg body weight). For each group, 8 micewere sacrificed for weigh, lung tissues and blood, 6 mice were anesthetized and detect lung morphometric analyses (repeat two times). All 56 mice were used in this study. Blood was collected from the right ventricle and centrifuged at 3000 rpm for 10 min at 4 °C. Then, the supernatant was stored at − 80 °C for further analysis. The lung tissues were harvested and collected as described below.

### Caffeine concentration in serum

The concentration of caffeine in serum was assessed by a standardized sandwich enzyme-linked immunosorbent assay (ELISA) kit (Biovision Inc., CA, USA) according to the manufacturer’s protocol. The ELISA plate was read at 450 nm and analyzed based on the standards.

### A_2A_R expression in lung tissues

Immunohistochemistry and western blotting were used to determine A_2A_R expression in lung tissues. For immunohistochemistry, five-micrometer sections of paraffin-embedded lung tissue were deparaffinized in xylene and rehydrated with graded ethanol solutions. Then, the sections were blocked in PBS containing 0.5% Triton X-100 for 15 min and pretreated with 5% bovine serum albumin for 30 min. The A_2A_R antibody (1:100) (Abcam, Cambridge, UK) was diluted in 10 mM PBS, and a total of 50 μl was added to the sections (overnight at 4 °C). The next day, the sections were treated with secondary antibodies (1:500, Biosharp Life Science, Hefei, China) (1 h at room temperature) and stained with 3,3′-diaminobenzidine (DAB) (30 s at room temperature). The western blot assay used to quantify A_2A_R expression is described below.

### Assessment of lung inflammation

Immunohistochemistry and western blotting were used to determine COX-1, COX-2 and MPO expression in lung tissues. The process was described above. The following primary antibodies were used: COX-1 (1:100); COX-2 (1:100) (Cell Signaling Technology, MA, USA); and MPO (1:50) (Abcam, Cambridge, UK). To measure MPO expression in the alveoli, MPO-positive cells were counted in a high-power field (HPF). A minimum of 12 nonoverlapping fields were examined in each group. The western blot analysis of COX-1, COX-2, TNF-α and IL-1β (Cell Signaling Technology, MA, USA) expression is described below.

### Assessment of oxidative stress in lung tissues

Peroxides are unstable indicators of oxidative stress in cells that decompose to form reactive compounds, such as malondialdehyde (MDA). Superoxide dismutase (SOD) and glutathione (GSH) are essential antioxidants that can scavenge superoxide anion free radicals and protect cells from hyperoxia-induced damage. The concentration of MDA and the activities of SOD and GSH in lung tissues were assessed by commercially available kits (Beyotime Biotechnology, Shanghai, China).

### Lung histological and morphometric analyses

Six mice from each group were sacrificed, and the right bronchus was ligated. The left lungs were perfused with 4% paraformaldehyde (PFA) at 20 cmH_2_O pressure via an intravenous needle inserted into the trachea. The left lungs were postfixed in 4% PFA for 48 h before the sections were embedded in paraffin and sectioned into 4-μm sections. The sections were stained with hematoxylin and eosin (HE) (Solarbio Science & Technology, Beijing, China) for morphometric analysis by microscopy (Nikon, Japan). The radial alveolar count (RAC), mean linear intercept (MLI) and mean alveolar diameter (MAD) were measured to quantify the interalveolar distance, as reported in previous studies [27]. MAD was calculated as the average alveolar diameter. MLI represents the volume-to-surface ratio of alveoli and was determined by drawing five lines in each field and dividing the length of each line by the number of alveoli that intercepted the line. RAC was obtained by drawing a line from the center of terminal bronchioles to the nearest connective tissue septum and counting the number of the alveoli on the line. All images were assessed by investigators blinded to the experimental groups.

### Assessment of SFTPC expression in lung

Immunofluorescence and western blotting were carried out for surfactant protein-C (SFTPC) expression in the lung. The immunofluorescence process was similar with immunohistochemistry. The antibodies were as follows: SFTPC antibody (1:100) (Abcam, Cambridge, UK), AlexaFluor 488 donkey anti-rabbit secondary antibodies (1:500, Biosharp Life Science, Hefei, China). The nucleus is shown by DAPI. The western blot assay used to determine SFTPC expression is described below.

### Analysis of inflammasome and NF-κB pathway protein expression in lung

Frozen lung tissues were used for protein assays. Protein lysates were obtained using RIPA lysis buffer supplemented with phosphatase inhibitors and phenylmethylsulfonyl fluoride (PMSF), ultrasonication for 5 s three times, and centrifugation at 12000 g for 20 min at 4 °C. Protein concentrations were measured by a Pierce™ BCA Protein Assay Kit. Thirty micrograms of protein were subjected to SDS-PAGE on 4–20% Tris-glycine gels (Bio-Rad, USA) and then electro-transferred to PVDF membranes. The membranes were blocked in 5% nonfat milk supplemented with TBST and incubated with primary (overnight at 4 °C) and secondary antibodies (2 h at room temperature). Antibodies against Caspase-1 (1:1000), SFTPC (1:1000), A_2A_R (1:1000) (Abcam, Cambridge, UK), NLRP3 (1:1000), ASC (1:1000), COX-1 (1:1000), COX-2 (1:1000), Caspase-3 (1:1000), NF-κB (1:1000), phosphor-NF-κB (1:1000), IκBα (1:1000), phosphor-IκBα (1:1000), TNF-α (1:1000), IL-1β (1:1000) (Cell Signaling Technology, MA, USA), and GAPDH (1:3000) (Bioworld Technology, Inc., Nanjing, China) were used. The protein bands were detected by Pierce™ ECL western blotting substrate (Thermo Fisher Scientific, Logan, USA) and visualized by a ChemiDocXRS^+^ Imaging System (Bio-Rad, USA).

### Measurement of cell apoptosis in lung

The TUNEL method is used to measure apoptotic DNA fragmentation. The In Situ Cell Death Detection Kit (Roche, South San Francisco, CA, USA) was used according to the manufacturer’s protocol. Five-micrometer sections of paraffin-embedded lung tissue were deparaffinized in xylene and rehydrated through graded ethanol solutions. Then, the sections were treated with 20 μg/ml proteinase K (15 min at 37 °C) and rinsed with PBS three times. A total of 50 μl of TUNEL reaction mixture was incubated with the slides in a dark humidified chamber (1 h at 37 °C). Then, the sections were treated with DAPI (5 min at room temperature) and rinsed with PBS three times. Apoptotic cells emitted strong green fluorescence from the nucleus according to the manufacturer’s protocol. The images were analyzed by ImageJ software. The results are shown as the percent of apoptotic cells.

### Statistical analysis

The experiments were performed in triplicate and repeated a minimum of three times. The data are presented as the mean ± SD and were analyzed by one-way analysis of variance (ANOVA) followed by Tukey’s post hoc test and Dunnett post hoc test versus the NO group (equal variance) or Dunnett T3’s post hoc test (unequal variance) for multiple comparisons. Correlation analyses were performed by Spearman’s rank correlation. Statistical analysis was carried out by SPSS Statistics version 19.0 (SPSS Inc., Chicago, IL) or GraphPad 6.0 (GraphPad Software, San Diego, CA, USA). Values of *P* < 0.05 versus the indicated group were considered statistically significant.

## Results

### The concentration of caffeine in serum

**Fig. 1:**
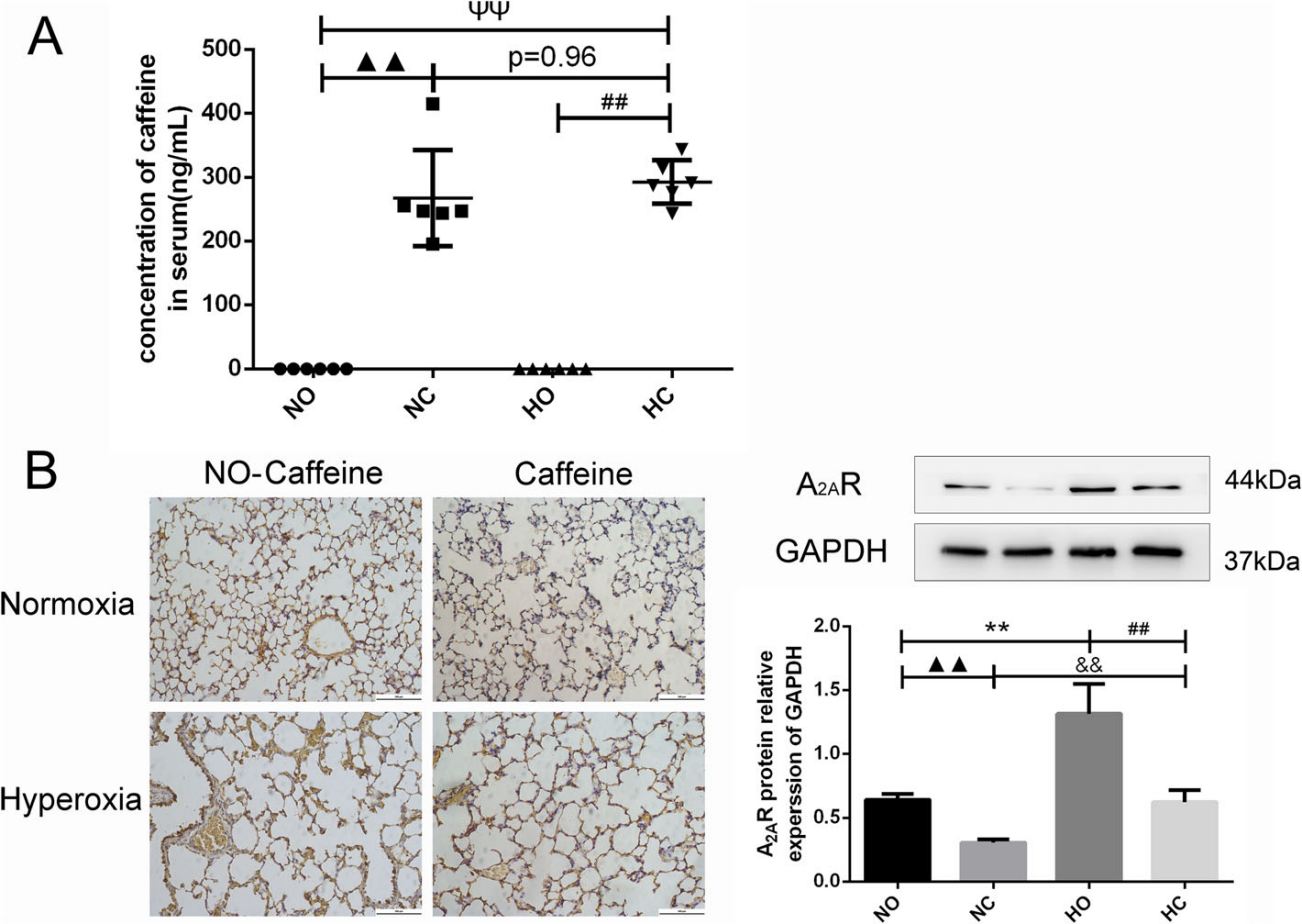
The concentration of caffeine in serum and expression of A_2A_R in lung. Caffeine treatment significantly increased the concentration of caffeine in serum and decreased the A_2A_R protein level in the lung. **a** The concentration of caffeine in serum as determined by ELISA. **b** The expression of A_2A_R in lung tissue as determined by IHC staining (light microscopy, × 200) and western blotting. Scale bars = 100 μm. The values are shown as the mean ± SD; *n* = 6 mouse/group. ^▲▲^*P* < 0.05 Normoxia (NO) group versus Normoxia +Caffeine (NC) group,^ΨΨ^*P* < 0.01 NO group versus Hyperoxia + Caffeine (HC) group, **P < 0.01 HO group versus NO group, ^##^*P* < 0.01 HC group versus HO group., ^&&^*P* < 0.01 HC group versus NC group

The caffeine level in plasma was detected by ELISA on P14. As shown in Fig. 1a, the level was below the detection limit in the Normoxia and Hyperoxia groups, while the level ranged from 195.01 ng/mL to 414.60 ng/mL when caffeine (1 g/L) was added to the water of nursing mothers. There was a significant difference in the groups administered caffeine and the groups administered pure water, and there was no difference in the caffeine concentration of the Normoxia + Caffeine (267.19 ± 75.40 ng/mL) and Hyperoxia + Caffeine (292.75 ± 34.02 ng/mL) group.

### The A_2A_R level in neonatal mouse lung

The A_2A_R level in the lung was detected by immunohistochemistry and western blotting. The expression of A_2A_R in the lung was increased in hyperoxia-exposed mice compared with normoxia-exposed mice, as shown in Fig. 1b. Caffeine is an adenosine receptor antagonist, and the A_2A_R level of the Normoxia + Caffeine group was lower than that in the normoxia control group. A_2A_R expression was decreased in the Hyperoxia + Caffeine group compared with the Hyperoxia group, and there was no significant difference in the A_2A_R levels of the Normoxia group and the Hyperoxia + Caffeine group. These results indicated that caffeine acts as an A_2A_R antagonist and can affect A_2A_R expression in lung cell.

### Caffeine decreased oxidative stress in neonatal mouse lung

**Fig. 2:**
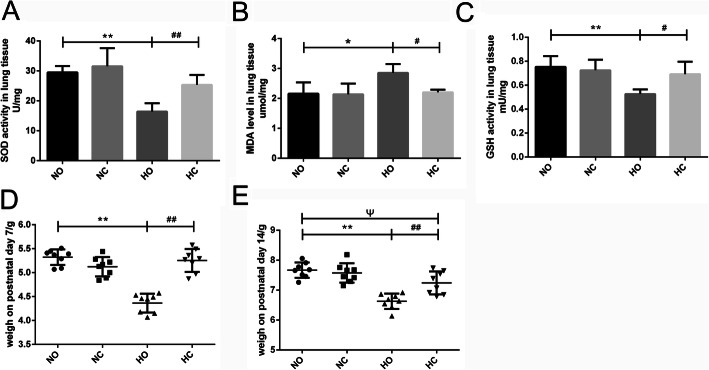
Effect of caffeine on the oxidative stress in lung and body weight of neonatal mice. After 14 days of high oxygen exposure, the SOD activity and GSH activity were decreased, and the MDA level was increased in the Hyperoxia (HO) group compared with the Normoxia (NO) group. Caffeine treatment reversed the hyperoxia-induced changes in these three oxidative indicators. **a** SOD activity in lung tissue. **b** MDA levels in lung tissue. **c** GSH activity in lung tissue. *n* = 6 mouse/group. **d** The body weight on postnatal day 7. (E) The body weight on postnatal day 14. *n* = 8 mice/group. *P < 0.05, **P < 0.01 HO group versus NO group, ^#^*P* < 0.05, ^##^*P* < 0.01 Hyperoxia + Caffeine (HC) group versus HO group, ^Ψ^*P* < 0.05 NO group versus HC group

The expression of oxidative stress in lung tissue was detected by available kits. As shown in Fig.2a-c, SOD and GSH activity were decreased and MDA level was distinctly increased in the Hyperoxia group compared with Normoxia group. Under caffeine treatment, MDA level decreased, and SOD and GSH activity increased. These results showed that caffeine treatment partly decreased the level of oxidative stress in HALI mice.

### Body weight of neonatal mice

After 7 days of 75% oxygen exposure, the body weight of mice was significantly decreased compared with the Normoxia group. However, body weight was increased in the Hyperoxia + Caffeine group compared with the Hyperoxia group at P7 (Fig. 2d). Similar changes in body weight were observed on P14 (Fig. 2e), but cannot completely reversed. These results showed that caffeine could increase the weight of mice after exposure to high oxygen.

### Effect of caffeine on the alveolar simplification of lung tissue

**Fig. 3:**
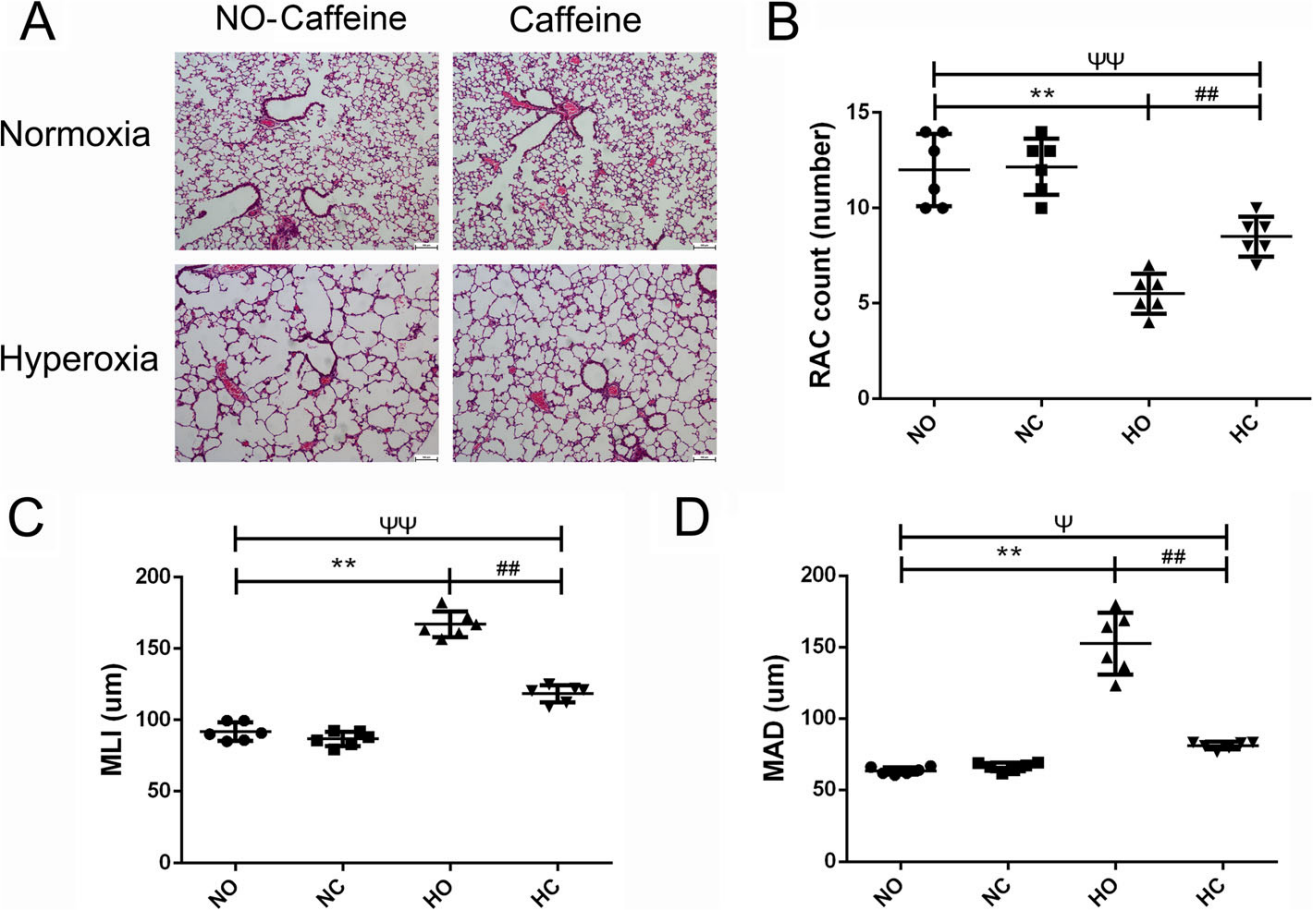
Effect of caffeine on pulmonary alveolar simplification in lung. Hyperoxia exposure led to marked alveolar simplification as shown by the HE staining in the images and by the assessment of RAC, MLI and MAD. Caffeine treatment improved the hyperoxia-induced impairment of alveolar growth. **a** Representative HE staining (light microscopy, × 100) of lung tissue slides from each group. Scale bars = 100 μm. **b** Semiquantitative pathology determination of RAC in lung tissues. **c** Semiquantitative pathology determination of MLI in lung tissues. **d** Semiquantitative pathology determination of MAD in lung tissues. The values are the mean ± SD; *n* = 6 mice/group. **P < 0.01 Hyperoxia (HO) group versus Normoxia (NO) group, ^##^*P* < 0.01 Hyperoxia + Caffeine (HC) group versus HO group, ^Ψ^*P* < 0.05, ^ΨΨ^*P* < 0.01 NO group versus HC group

The lung development is shown in Fig. 3 by HE staining. The lung in the Normoxia group showed intact lung structures with a normal alveolar epithelium, uniform alveolar septum and clear arrangement of airway and blood vessels. And in the Normoxia + Caffeine group, the lung sections had a similar structure to that in the Normoxia group. After 14 days of high oxygen exposure, we observed a markedly simplified alveolar appearance in the lungs of mice exposed to hyperoxia (Fig. 3a). In per unit area, the alveolar volume increased, the number of alveoli decreased, and the alveolar interval increased, as represented by the decrease in RAC and the increase in MLI and MAD (Fig. 3b-d). These results suggested that exposure to high oxygen levels increased alveolar injury, resulted in alveolar simplification and delayed lung development. Alveolar simplification was significantly improved in the Hyperoxia + Caffeine group compared with the Hyperoxia group. Moreover, the alveolar septum was thinner in the Hyperoxia + Caffeine group than that in Hyperoxia group. Compared with the Hyperoxia group, the Hyperoxia + Caffeine group had increased RAC and decreased MLI and MAD (Fig. 3). These results indicated that caffeine treatment could restore hyperoxia-induced changes in alveolar formation.

### Caffeine decreased inflammation infiltration in the mouse lung

**Fig. 4:**
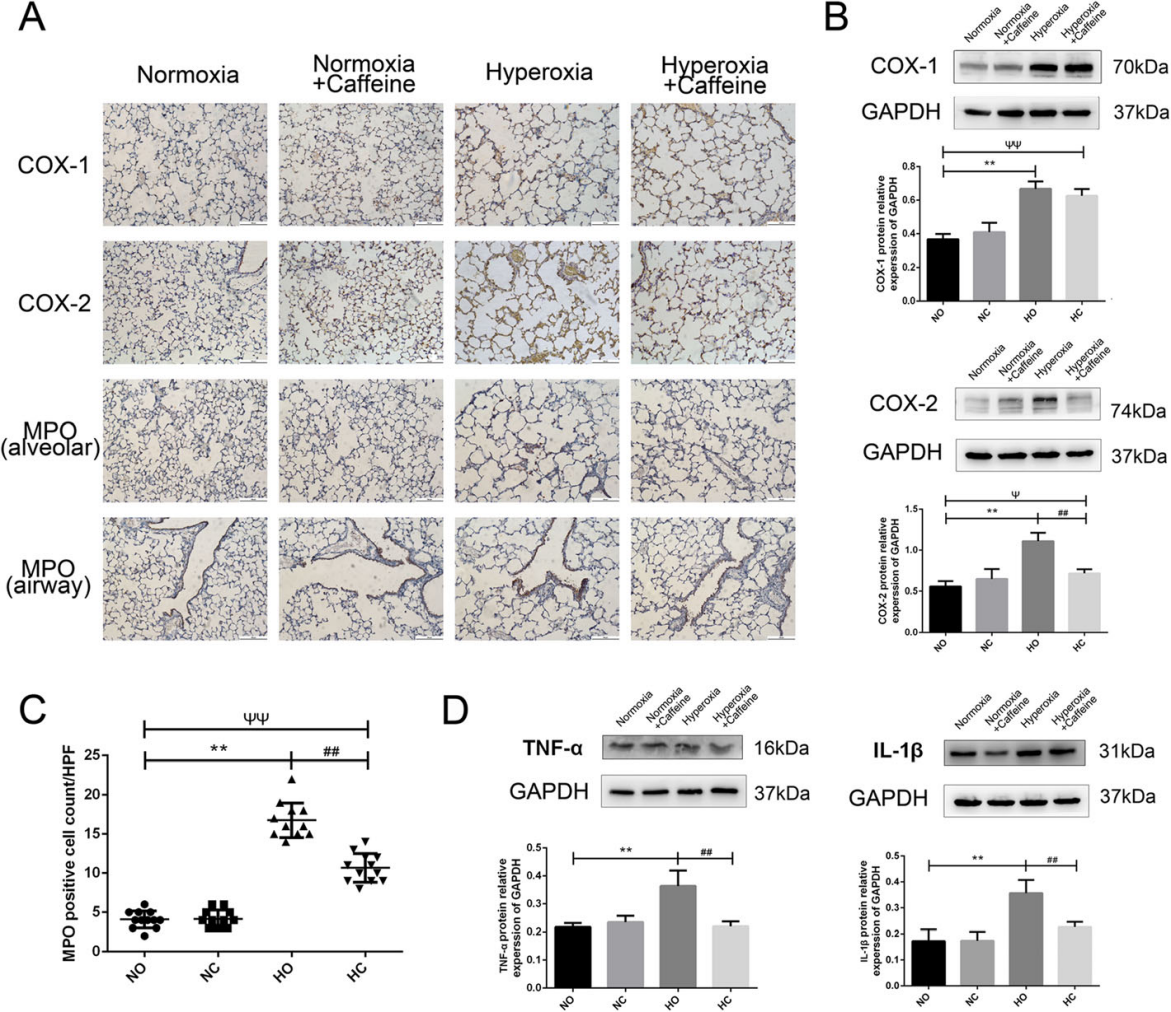
Caffeine reduced the inflammatory reaction in neonatal mouse lung. Hyperoxia exposure could increase the expression of COX-1, COX-2, MPO, IL-1β and TNF-α in the lung, as shown, and caffeine treatment could decrease the levels of COX-2, MPO, IL-1β and TNF-α. **a** The expression of COX-1, COX-2 and MPO protein in lung as determined by IHC staining (light microscopy, × 100). Scale bars = 100 μm. **b** The protein levels of COX-1 and COX-2 in the lung as determined by western blotting. **c** The number of MPO-positive cells in the lung alveolar space in a high-power field (HPF). **d** The protein levels of IL-1β and TNF-α in the lung as determined by western blotting. The values are the mean ± SD; n = 6 mice/group. **P < 0.01 Hyperoxia (HO) group versus Normoxia (NO) group, ^##^*P* < 0.01 Hyperoxia + Caffeine (HC) group versus HO group, ^Ψ^*P* < 0.05, ^ΨΨ^*P* < 0.01 NO group versus HC group

The expression of inflammatory cytokines in the lung was detected by immunohistochemistry and western blotting. We detected the levels of COX-1, COX-2, MPO and TNF-α and IL-1β. As shown in Fig. 4a-b, the levels of COX-1 and COX-2 were significantly higher in Hyperoxia group than that in the Normoxia group. Caffeine decreased the protein expression of COX-2, but not that of COX-1. MPO, a characteristic marker of myeloid cells, was found on neutrophils, monocytes and macrophages. The MPO level can reflect the activation of neutrophils. As shown in Fig. 4a, MPO-positive cell were found in the lung alveolar space and respiratory airway. The number of MPO-positive cells was significantly increased in the Hyperoxia group, and caffeine decreased that on P14 (Fig. 4c).

We detected the expression of TNF-α and IL-1β protein in lung tissue. As shown in Fig. 4d, we observed increased TNF-α level in the lung of the Hyperoxia group compared with those of the Normoxia group. The caffeine treatment reduced TNF-α level, almost to that in Normoxia group. Similar change in the IL-1β level was found. These results suggest that caffeine ameliorated the inflammation reaction induced by hyperoxia in lung.

### Caffeine increased SFTPC expression in neonatal mice

**Fig. 5:**
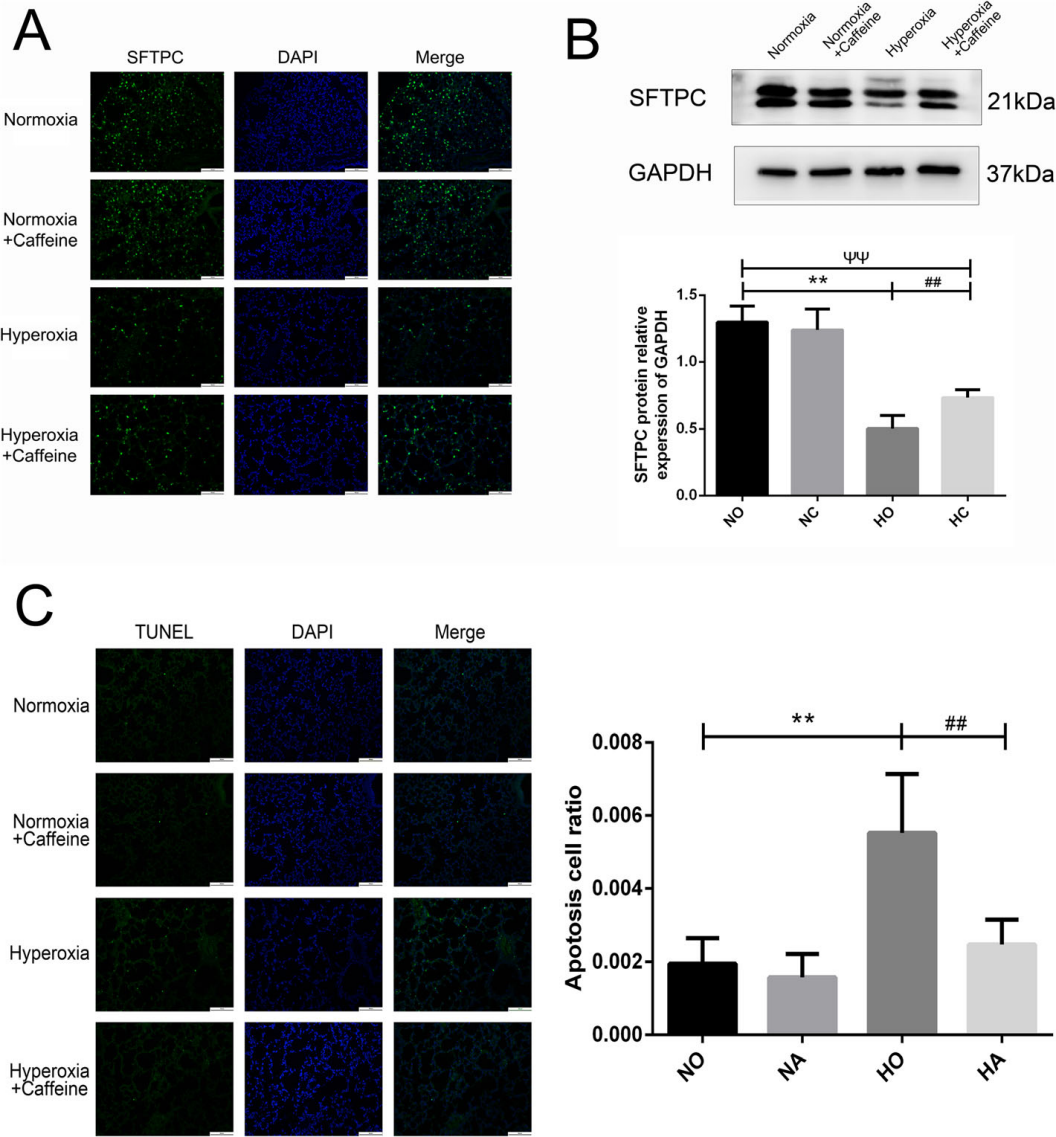
The effect of caffeine on SFTPC expression and apoptosis. After 14 days of high oxygen exposure, SFTPC protein expression was significantly decreased, and the number of apoptotic cells was increased. Caffeine treatment reversed these effects. **a** Representative SFTPC-positive cells in the lungs of each group as determined by IF staining (microscopy, × 200). Scale bars = 100 μm. **b** The protein level of SFTPC in lung as determined by western blotting. **c** Representative images and an analysis of TUNEL staining (microscopy, × 200). Scale bars = 100 μm. The values are the mean ± SD; n = 8 fields/group. **P < 0.01 Hyperoxia (HO) group versus Normoxia (NO) group, ^##^*P* < 0.01 Hyperoxia + Caffeine (HC) group versus HO group, ^▲^*P* < 0.05 NO group versus HC group,^ΨΨ^*P* < 0.01 NO group versus HC group

Type II alveolar epithelial cell is the stem cell in the newborn lung that can effectively differentiate into Type I alveolar epithelial cell, which constitute the alveolar structure involved in pulmonary respiratory function. SFTPC protein is the characteristic protein expressed in Type II alveolar epithelial cell. The level of SFTPC protein in the lung tissue was detected by immunofluorescence and western blotting. As shown in Fig. 5a, the SFTPC in the Normoxia group and the Normoxia + Caffeine group was similar. However, it was lower in Hyperoxia group compared with Normoxia group. Caffeine treatment could reverse the hyperoxia-induced reduction in SFTPC expression to a level lower than that in Normoxia group. This finding revealed that caffeine had a positive effect on the quantity of Type II alveolar epithelial cells in HALI mouse lung.

### Caffeine decreased the cell apoptosis associated with hyperoxia exposure in neonatal mice

The TUNEL method was used to detect cell apoptosis in mouse lungs. As shown in Fig. 5b, there was almost no cell apoptosis in the Normoxia and Normoxia + Caffeine group. After 14 days of 75% oxygen exposure, cell apoptosis was significantly increased in the Hyperoxia group compared with Normoxia group. After treatment with caffeine, the hyperoxia-induced cell apoptosis was markedly decreased, similar to the level in the Normoxia group.

### Caffeine decreased the expression of NLRP3 inflammasome proteins and the phosphorylation of NF-κB pathway in the lungs

**Fig. 6:**
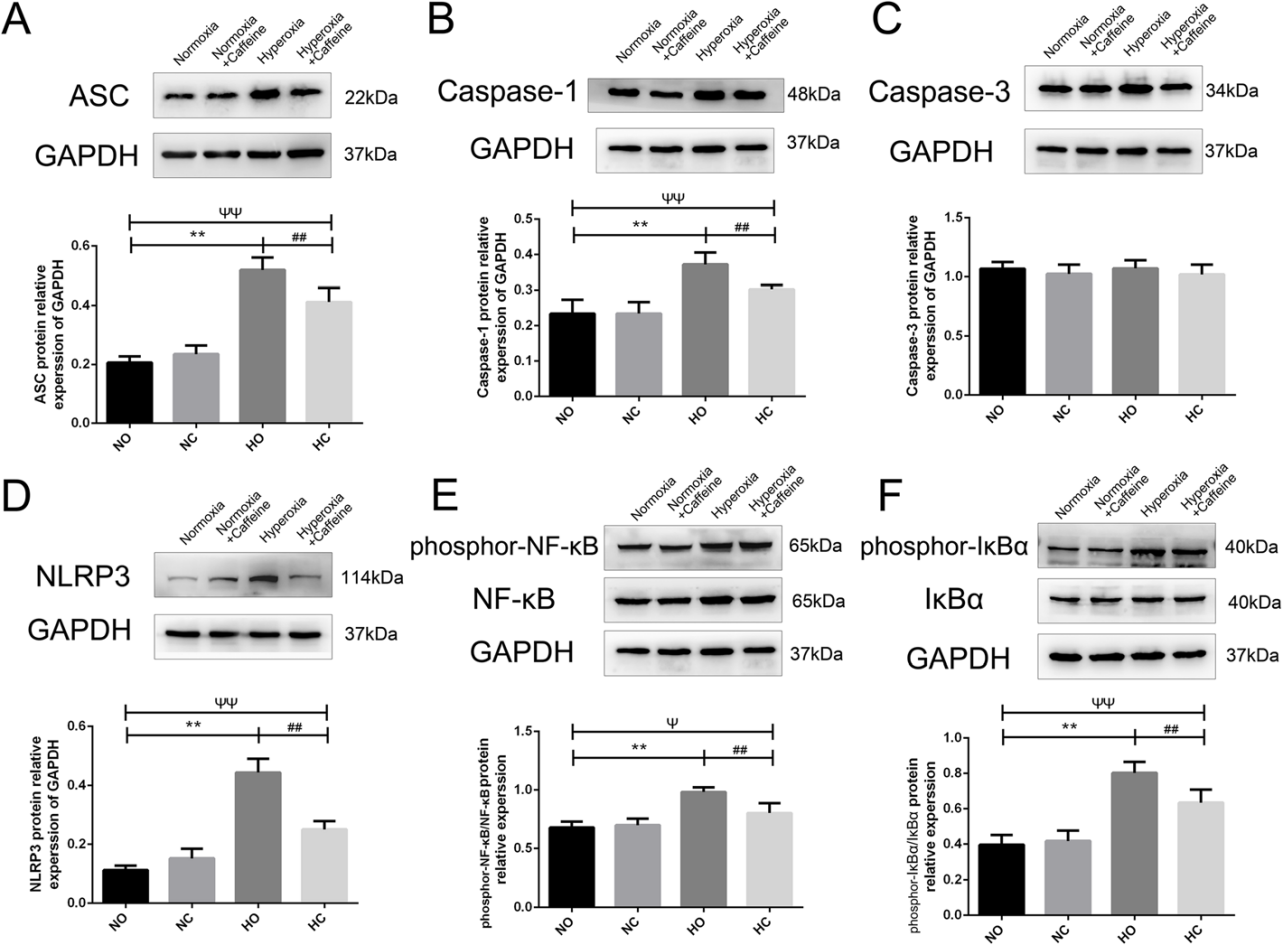
The effect of caffeine on the NF-κB pathway and inflammasome proteins in neonatal mouse lung. Hyperoxia exposure led to the activation of the NF-κB pathway and inflammasome proteins. Caffeine treatment ameliorated this hyperoxia-induced change. **a** Representative image and semiquantitative analysis of ASC protein expression in lung tissue. **b** Representative image and semiquantitative analysis of Caspase-1 protein expression in lung tissue. **c** Representative image and semiquantitative analysis of Caspase-3 protein expression in lung tissue. **d** Representative image and semiquantitative analysis of NLRP3 protein expression in lung tissue. **e** Representative image and semiquantitative analysis of phosphor-NF-κB and NF-κB protein expression in lung tissue. **f** Representative image and semiquantitative analysis of phosphor-IκBα and IκBα protein expression in lung tissue. GAPDH was the loading control. The values are the mean ± SD; n = 6 mice/group. **P < 0.01 Hyperoxia (HO) group versus Normoxia (NO) group, ^##^*P* < 0.01 Hyperoxia + Caffeine (HC) group versus HO group, ^Ψ^*P* < 0.05, ^ΨΨ^*P* < 0.01 NO group versus HC group

To evaluate the effect of caffeine on inflammasome proteins in the lung, we examined lung tissues by western blot analyses. We quantified the expression of ASC, NLRPS, Caspase-1 and Caspase-3 in the lungs. As shown in Fig. 6a-d, ASC expression was increased in the Hyperoxia group compared with the Normoxia group, and this effect could be partly reversed by the caffeine intervention. Similar changes were found in the protein expression of NLRP3 and Caspase-1. There was no significant difference in the protein expression of Caspase-3 among the mouse treatment groups.

Next, we investigated whether caffeine could change the phosphorylation levels of members of the NF-κB pathway in neonatal mice exposed to hyperoxia. As shown in Fig. 6e-f, hyperoxia exposure could activate the NF-κB pathway as phosphor-IκBα and phosphor- NF-κB were markedly increasing in the Hyperoxia group. Caffeine could partly decrease the activation of the NF-κB pathway. These results indicate that caffeine has a positive effect on the formation of the inflammasome and the activation of the NF-κB pathway in mouse lungs after hyperoxic exposure.

### Correlations among serum caffeine concentration, A_2A_R protein and lung injury outcomes

**Fig. 7:**
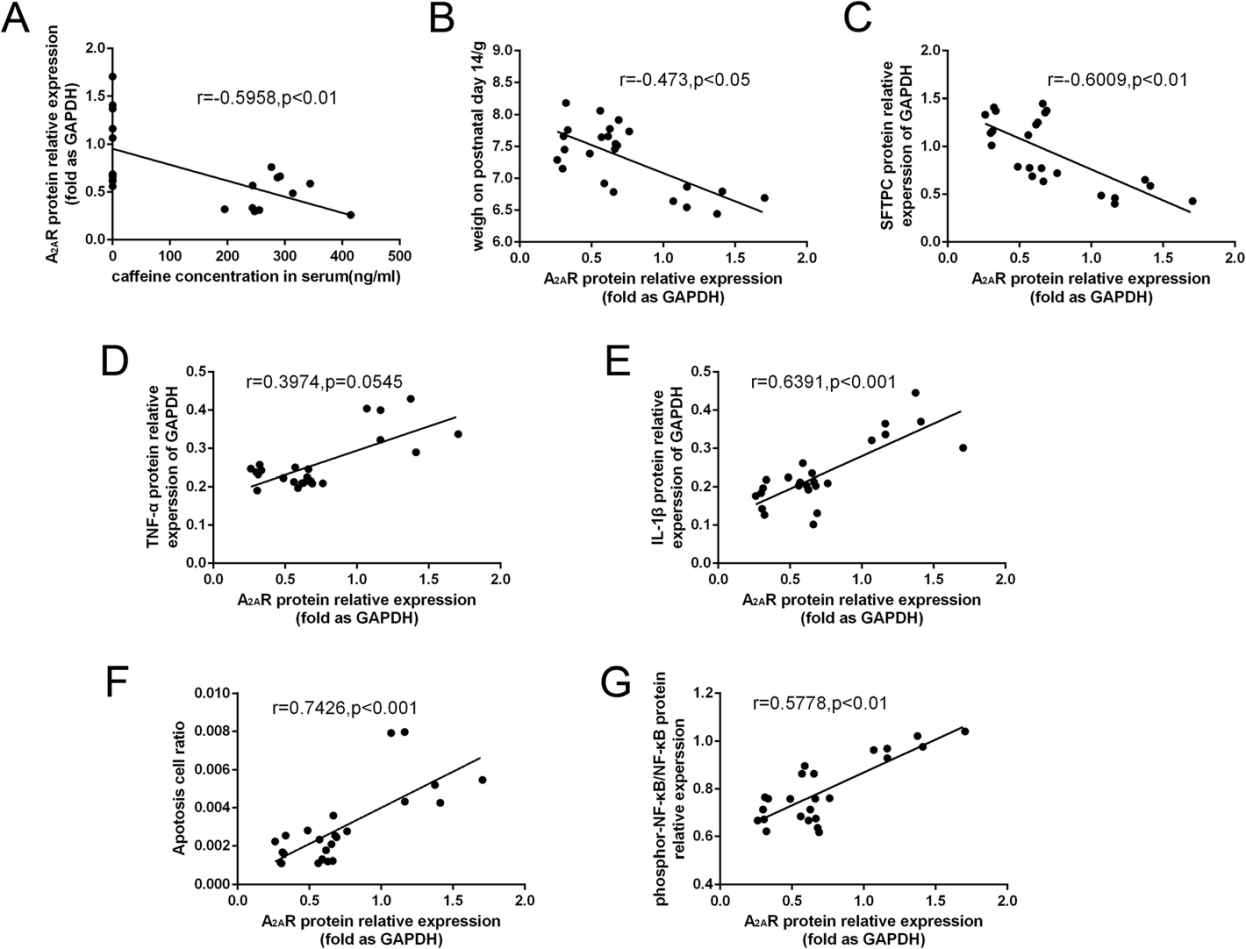
The correlations among serum caffeine concentration, A_2A_R protein and lung injury outcomes. **a** The correlation of serum caffeine concentration and A_2A_R protein, *n* = 24, r = − 0.5958, *P* < 0.01. **b** The correlation of A_2A_R protein and mice body weight on P14, *n* = 24, r = − 0.473, *P* < 0.05. **c** The correlation of A_2A_R protein and SFTPC protein, *n* = 24, r = − 0.6009, *P* < 0.01. **d** The correlation of A_2A_R protein and TNF-α, *n* = 24, r = 0.3974, *P* = 0.0545. **e** The correlation of A_2A_R protein and IL-1β, *n* = 24, r = 0.6391, *P* < 0.001. **f** The correlation of A_2A_R protein and apotosis cell, *n* = 24, r = 0.7426, *P* < 0.001. **g** The correlation of A_2A_R protein and phosphor-NF-κB, *n* = 24, r = 0.5778, *P* < 0.01

Finally, we determined the association between caffeine concentration, A_2A_R protein and lung injury outcomes. As shown in Fig. 7a, the caffeine concentration was negatively correlated with A_2A_R protein (*P* < 0.01). Moreover, in the analysis of A_2A_R protein and other lung injury indexes, there was a negative correlation between A_2A_R protein and mice body weight on P14, SFTPC protein, and a positive correlation between A_2A_R protein and apoptosis cell, pro-inflammatory cytokines and phosphor-NF-κB protein (Fig. 7b-g).

## Discussion

BPD is a common clinical complication in premature infants. Prolonged exposure of immature lungs to high levels of oxygen has been reported to be one of the main reason in the development of BPD [28]. The early use of caffeine has been clinically confirmed to effectively reduce the incidence of BPD [29], but the specific mechanism remains unclear and has not been thoroughly studied. We established a BPD model in neonatal mice by exposing mice to 75% oxygen. Caffeine was administered to nursing mice, which resulted in a distinct effective caffeine content in the blood and in antagonistic, competitive binding with A_2A_R in the lung cells of neonatal mice.

We found that caffeine effectively reduced the oxidative stress, improved weight gain, promoted alveolar development, reduced inflammatory infiltration and lung cell injury induced by hyperoxia in mice. Moreover, caffeine could decrease the apoptosis and promote the proliferation of type II alveolar epithelial cells. The mechanism may be related to the reduction of NLRP3 inflammasome formation and the down-regulation of the NF-κB pathway.

Caffeine is a non-selective adenosine A_1_ and A_2_ receptors inhibitor, which stimulate respiratory center, increase CO2 sensitivity, and enhance respiratory muscle contractility. A number of studies, such as Dayanim, Teng, Rath, and Nagatomo [23, 30–32], used intraperitoneal injections to reach the effective concentration in neonatal mice. In Teng’s study, caffeine was reported as a non-selective phosphodiesterase inhibitor [23]. In Endesfelder’s research, caffeine was reported as a potent antioxidant [33]. One of the advantages of our experiment was that we added caffeine to the drinking water of nursing mice. By giving the mother mice drinking water containing 1 g/L of caffeine, the mice were able to achieve a serum caffeine concentration of 195–414.6 ng/mL, same reported as previously [26]. So the concentration above in HC and NC group could effectively block A_2A_R, the target of caffeine in the lung.

High oxygen exposure lung injury is the most common model in newborn pups for BPD, which can cause alveolar simplification, vascular malformation and pulmonary fibrosis in lung. Compare the lung development stage in mice and human, we exposed the neonatal mice to 75% oxygen on postnatal day 0–14, which represents the saccular and alveolar stage in mice [34, 35]. Moreover, exposure to high levels of oxygen inevitably leads to the production of excess oxygen radicals [6]. Our study found that MDA level was increased and that SOD and GSH activity decreased in neonatal mice after hyperoxic exposure, which confirmed the effective establishment of a hyperoxic lung injury model. Compared with normoxic conditions, hyperoxic exposure resulted in decreased postnatal weight gain, distinct alveoli simplification, increased inflammatory protein expression, marked inflammatory cell infiltration, and increased apoptotic cells in mice. After the caffeine intervention, the weight gain of neonatal mice was significantly improved, nearly the same as that of the mice in the NO group on P7, indicating that caffeine could help improve hyperoxia-induced weight loss. The number of alveoli per unit lung area was significantly increased, RAC increased, MAD and MLI decreased in the HC group mice compared with the HO group mice, which was similar to Teng’s report [23]. Moreover, compared with the HO group, the HC group exhibited deceased TNF-α and IL-1β expression and decreased neutrophil infiltration. The TUNEL results also showed a marked reduction in lung cell apoptosis following caffeine treatment. The above data indicated that caffeine could reduce the severity of hyperoxia-induced lung injury, affect the inflammatory cytokines and alveolar injury degree. And this result was similar with that in Weichelt’s study [36].

COX is a rate-limiting enzyme induced by inflammation or tissue injury that catalyzes the reaction that changes arachidonic acid into prostaglandin (PG) and regulates various physiological and pathological processes [37]. COX-1 is widely found on the surface of blood vessels, plays a role in the generation of PG and protects cells and tissues. Additionally, COX-2 mainly mediates the release of various proinflammatory mediators after inflammatory stimulation [38, 39]. In Teng’s study, COX-2 level was higher in hyperoxia group on P10 and P21, and that could be attenuated by caffeine, but no difference in COX-1 level [23]. Our results indicate that COX-1 and COX-2 were significantly increased in lung tissues after hyperoxia exposure andcaffeine treatment significantly decreased COX-2 expression but did not significantly change COX-1 expression. This result indicated that caffeine intervention did not affect the expression of COX-1 in the lung, which may be related to the proliferation and expansion of pulmonary blood vessels and the increase in pulmonary interstitial cells after hyperoxic exposure. Caffeine did not affect the hyperoxic vascular hyperplasia caused by hyperoxic exposure. The significant decrease in COX-2 indicated that caffeine could reduce the expression of COX-2 in lung tissue, thereby reducing the expression of various downstream inflammatory mediators, which was consistent with the decrease in the inflammatory factors above. Moreover, the difference on COX-1 protein between our data and Teng’s, maybe related to the oxygen exposure time and the experimental animal, which should be studied in more research.

Type II alveolar epithelial cell is stem cell in the lung that account for almost two-thirds of alveolar epithelial cells [40]. SFTPC is the characteristic protein of type II alveolar epithelial cells. Currently, there are many controversies regarding SFTPC expression and high oxygen exposure. In Johnston’s study, 95% oxygen exposure on type II alveolar epithelial cells significantly decreased the expression of SFTPC [41]. In Zhang’s report, high oxygen exposure for 14 days significantly increased the expression of SFTPC and inhibited the transformation of type II alveolar epithelial cells into type I alveolar epithelial cells [42]. In Jin’s study, SFTPC increased from P3 to P7 and decreased from P10 to P14 after hyperoxic exposure [43]. Our data showed a distinct reduction in SFTPC expression in the lung after 14 days of high oxygen exposure using immunofluorescence and western blotting. This reduction may be associated with impaired alveolar development. Hyperoxia reduced the proliferation and increased the apoptosis of pulmonary cells, and the number of type II alveolar epithelial cells was significantly reduced in newborn mice exposed to hyperoxia compared with newborn mice exposed to room air. The increasing expression of SFTPC in the HC group suggested that type II alveolar epithelial cells could recover or regain function after caffeine intervention.

**Fig. 8:**
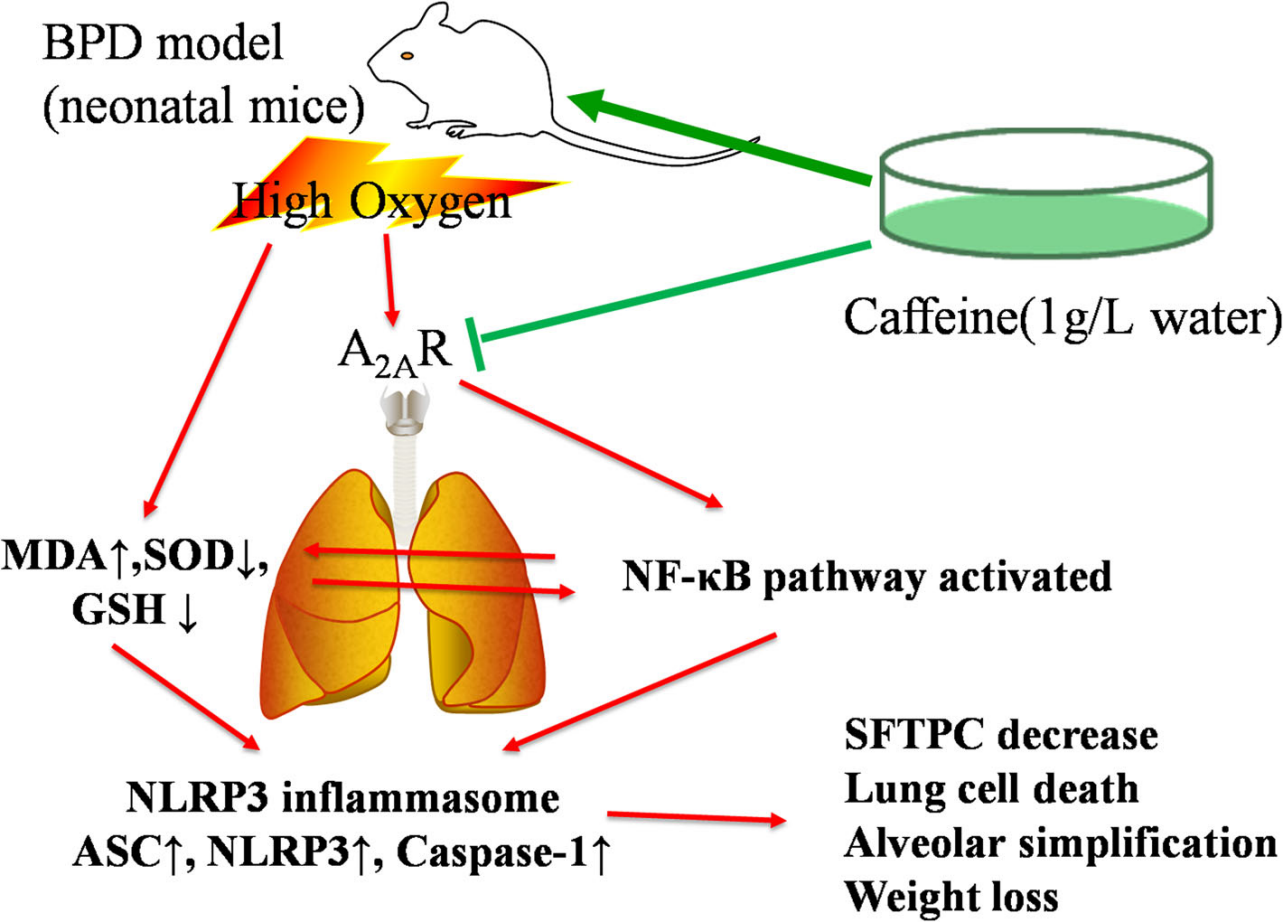
Potential mechanisms of the caffeine treatment-mediated mitigation of hyperoxia-induced lung injury in neonatal mice. High oxygen exposure led to the release of reactive oxygen species (ROS) in newborn mice lung, activated the NF-κB pathway and NLRP3 inflammasomes release, resulted in lung cell damage and alveolar simplification. Caffeine could antagonize A_2A_R and inhibit the NF-κB pathway, reduce the activation of NLRP3 inflammasome, and alleviate the alveolar injury

In Dayanim’s study, the use of caffeine would induce the alveolar apoptosis, in contrast to Teng, Nagatomo, Jing and our results [23, 30–32, 44]. Now, caffeine is the first line of prevention and treatment for apnea, which can reduce the severity of lung injury. As in animal studies, most articles shown protective effect and regarding the mechanism on hyperoxia-induced lung injury in neonatal mice. Many studies had focused on the TGF-beta pathway. Tatler found that caffeine inhibited the fibrosis response induced by the TGF-beta pathway in lung fibroblasts and alpha-SMA formation [45]. Fehrholz found that caffeine affects airway remodeling in the lung via the TGF-beta1-Smad pathway [24]. Teng found that caffeine was a non-selective phosphodiesterase inhibitor, reduced pulmonary inflammation and apoptosis by inhibiting endoplasmic reticulum (ER) stress and affected mitochondrial function [23]. Endesfelder found that caffeine appeared to be a potent antioxidant and reduced hyperoxia-induced pulmonary oxidative stress response in rat model [33]. In our study, as IL-1β and TNF-α level were increased, we studied the role of the NF-κB pathway and NLRP3 inflammasome in the protective effect of caffeine against hyperoxia-induced lung injury. We found that caffeine intervention in HALI neonatal mice resulted in a significant reduction inNLRP3 inflammasome proteins and the activation of the NF-κB pathway. This finding suggested that the formation of the pulmonary NF-κB pathway and NLRP3 inflammasome was affected by the competitive and antagonistic interaction with the lung cell A_2A_ receptor, thereby reduced the expression of inflammatory cytokines, decreasing the apoptosis of alveolar epithelial cells, and improving the survival and growth of lung cells in early lung development (Fig. 8).

A_2A_R protein is recognized as an important mediator of inflammatory and immune responses. These findings above had showed that the treatment with caffeine markedly reduced the expression of pro-inflammatory cytokines, decreased the expression of NLRP3 inflammasome and NF-κB pathway. Then, we determined the association between A_2A_R protein and inflammatory indexes in hyperoxia mice model. We found that TNF-α, IL-1β and phosphor-NF-κB protein were positively correlated to A_2A_R protein in mice. Take together, our data strongly suggested that inhibition of A_2A_R could be an effective therapy for high oxygen exposed ALI mice. Further preclinical and clinical study need to address this potential.

Some limitations of our study are to be mentioned. (i) Our model used 75% high oxygen to establish the BPD model, which may only reflect part of the clinical manifestations. (ii) Although we found that the NF-κB pathway and NLRP3 inflammasome were increased in the model of hyperoxia-induced lung injury, caffeine was able to reduce this change. However, these data may not be mechanically linked. NLRP3 or NF-κB pathway inhibitors should added into the future investigations. (iii) Epithelial cells and fibroblasts play important roles in the BPD model, and it has been reported that the effects of caffeine on epithelial cells and fibroblasts are inconsistent [45], which needs to be further explored in a cell-based experiment. (iv) We added caffeine in mother dams’ diet at the dosage of 1 g/L, other administration mode and dosage, also Pulmonary performance on P14 or moreshould also be considered for more reliable evidences in clinic.

## Conclusions

In summary, our study suggested that postnatal caffeine administration can ameliorate lung injury after hyperoxia exposure by reducing the activation of the NF-κB pathway and inhibiting the formation of the NLRP3 inflammasome. Caffeine, as an A_2A_ receptor antagonist, reduced the pulmonary inflammatory infiltration and lung cell apoptosis, promoted type II alveolar epithelial cell development. Our study provided a new mechanism by which caffeine protects against hyperoxic lung injury, providing necessary evidence for caffeine treatment in BPD infants.

## Data Availability

The datasets used and/or analyzed during the current study are available from the corresponding author on reasonable request.

## References

[1] Principi N, Di Pietro G, Esposito S (2018). Bronchopulmonary dysplasia: clinical aspects and preventive and therapeutic strategies. J Transl Med.

[2] Kalikkot Thekkeveedu R, Guaman M, Shivanna B (2017). Bronchopulmonary dysplasia: a review of pathogenesis and pathophysiology. Respir Med.

[3] Hwang J, Rehan V (2018). Recent advances in Bronchopulmonary dysplasia: pathophysiology, prevention, and treatment. Lung.

[4] Wang J, Dong W. Oxidative stress and bronchopulmonary dysplasia. Gene 2018. 10.1016/j.gene.2018.08.031 30098433

[5] De Wijs-Meijler DP, Duncker DJ, Tibboel D, et al. Oxidative injury of the pulmonary circulation in the perinatal period: short- and long-term consequences for the human cardiopulmonary system. Pulmon Circ. 2017;7(1):55–66. 10.1086/689748 PMC5448552 28680565

[6] Kallet RH, Matthay MA (2013). Hyperoxic acute lung injury. Respir Care.

[7] Hummler JK, Dapaah-Siakwan F, Vaidya R (2017). Inhibition of Rac1 signaling Downregulates Inflammasome activation and attenuates lung injury in neonatal rats exposed to Hyperoxia. Dis Model Mech.

[8] Vaidya R, Zambrano R, Hummler JK (2017). Recombinant CCN1 prevents hyperoxia-induced lung injury in neonatal rats. Pediatr Res.

[9] Zhang L, Zhao S, Yuan L (2016). Knockdown of placental growth factor (PLGF) mitigates hyperoxia-induced acute lung injury in neonatal rats: suppressive effects on NF-kappaB signaling pathway. Int Immunopharmacol.

[10] Britt RD, Velten M, Tipple TE (2013). Cyclooxygenase-2 in newborn hyperoxic lung injury. Free Radic Biol Med.

[11] Rumzhum NN, Ammit AJ (2016). Cyclooxygenase 2: its regulation, role and impact in airway inflammation. Clin Exp Allergy.

[12] Khan AA, Alsahli MA, Rahmani AH. Myeloperoxidase as an Active Disease Biomarker: Recent Biochemical and Pathological Perspectives. Medical sciences. 2018;6(2). 10.3390/medsci6020033 PMC6024665 29669993

[13] Kesavardhana S, Kanneganti TD (2017). Mechanisms governing inflammasome activation, assembly and pyroptosis induction. Int Immunol.

[14] Yang Y, Wang H, Kouadir M (2019). Recent advances in the mechanisms of NLRP3 inflammasome activation and its inhibitors. Cell Death Dis.

[15] Groslambert M, Py BF (2018). Spotlight on the NLRP3 inflammasome pathway. J Inflamm Res.

[16] Mangan MSJ, Olhava EJ, Roush WR (2018). Targeting the NLRP3 inflammasome in inflammatory diseases. Nat Rev Drug Discov.

[17] Grailer JJ, Canning BA, Kalbitz M (2014). Critical role for the NLRP3 inflammasome during acute lung injury. J Immunol.

[18] Mizushina Y, Shirasuna K, Usui F (2015). NLRP3 protein deficiency exacerbates hyperoxia-induced lethality through Stat3 protein signaling independent of interleukin-1beta. J Biol Chem.

[19] Liao J, Kapadia VS, Brown LS (2015). The NLRP3 inflammasome is critically involved in the development of bronchopulmonary dysplasia. Nat Commun.

[20] Jobe AH (2017). Caffeine: a lung drug for all very low birth weight preterm infants?. Am J Respir Crit Care Med.

[21] Pedata F, Pugliese AM, Coppi E (2014). Adenosine A2A receptors modulate acute injury and neuroinflammation in brain ischemia. Mediat Inflamm.

[22] Kua KP, Lee SW (2017). Systematic review and meta-analysis of clinical outcomes of early caffeine therapy in preterm neonates. Br J Clin Pharmacol.

[23] Teng RJ, Jing X, Michalkiewicz T (2017). Attenuation of endoplasmic reticulum stress by caffeine ameliorates hyperoxia-induced lung injury. Am J Physiol Lung Cell Mole Physiol.

[24] Fehrholz M, Speer CP, Kunzmann S (2014). Caffeine and rolipram affect Smad signalling and TGF-beta1 stimulated CTGF and transgelin expression in lung epithelial cells. PLoS One.

[25] Zhao W, Ma L, Cai C (2019). Caffeine inhibits NLRP3 Inflammasome activation by suppressing MAPK/NF-kappaB and A2aR signaling in LPS-induced THP-1 macrophages. Int J Biol Sci.

[26] Zhang S, Zhou R, Li B (2017). Caffeine preferentially protects against oxygen-induced retinopathy. FASEB J.

[27] Wu QP, Chong L, Shao YY (2019). Lipoxin A4 reduces hyperoxia-induced lung injury in neonatal rats through PINK1 signaling pathway. Int Immunopharmacol.

[28] Shahzad T, Radajewski S, Chao C (2016). Pathogenesis of bronchopulmonary dysplasia: when inflammation meets organ development. Mol Cell Pediatr.

[29] Jain D, Bancalari E (2017). Prevention of bronchopulmonary dysplasia: current strategies. Chin J Contemp Pediatr.

[30] Dayanim S, Lopez B, Maisonet TM (2014). Caffeine induces alveolar apoptosis in the hyperoxia-exposed developing mouse lung. Pediatr Res.

[31] Rath P, Nardiello C, Morty RE (2017). A new target for caffeine in the developing lung: endoplasmic reticulum stress?. Am J Physiol Lung Cell Mol Physiol.

[32] Nagatomo T, Jimenez J, Richter J (2016). Caffeine prevents Hyperoxia-induced functional and structural lung damage in preterm rabbits. Neonatology.

[33] Endesfelder S, Strauss E, Scheuer T, et al. Antioxidative effects of caffeine in a hyperoxia-based rat model of bronchopulmonary dysplasia. Respir Res. 2019;20:88. 10.1186/s12931-019-1063-5 PMC6511176 31077204

[34] Berger J, Bhandari V (2014). Animal models of bronchopulmonary dysplasia. The term mouse models. Am J Physiol Lung Cell Mol Physiol.

[35] Nardiello C, Mižíková I, Morty R (2017). Looking ahead: where to next for animal models of bronchopulmonary dysplasia?. Cell Tissue Res.

[36] Weichelt U, Cay R, Schmitz T, et al. Prevention of hyperoxia-mediated pulmonary inflammation in neonatal rats by caffeine. Eur Respir J. 2013;41:966–73. 10.1183/09031936.00012412 22878872

[37] Goppelt-Struebe M (1995). Regulation of prostaglandin endoperoxide synthase (cyclooxygenase) isozyme expression. Prostaglandins Leukot Essent Fat Acids.

[38] Hla T, Bishop-Bailey D, Liu CH (1999). Cyclooxygenase-1 and -2 isoenzymes. Int J Biochem Cell Biol.

[39] Fukunaga K, Kohli P, Bonnans C (2005). Cyclooxygenase 2 plays a pivotal role in the resolution of acute lung injury. J Immunol.

[40] Brody JS, Williams MC (1992). Pulmonary alveolar epithelial cell differentiation. Annu Rev Physiol.

[41] Johnston LC, Gonzales LW, Lightfoot RT (2010). Opposing regulation of human alveolar type II cell differentiation by nitric oxide and hyperoxia. Pediatr Res.

[42] Zhang L, Zhao S, Yuan L (2016). Hyperoxia-mediated LC3B activation contributes to the impaired transdifferentiation of type II alveolar epithelial cells (AECIIs) to type I cells (AECIs). Clin Exp Pharmacol Physiol.

[43] Jin Y, Peng LQ, Zhao AL (2018). Hyperoxia induces the apoptosis of alveolar epithelial cells and changes of pulmonary surfactant proteins. Eur Rev Med Pharmacol Sci.

[44] Jing XG, Huang YW, Jarzembowski J, et al. Caffeine ameliorates hyperoxia-induced lung injury by protecting GCH1 function in neonatal rat pups. Pediatr Res. 2017;82:483–9. 10.1038/pr.2017.89 PMC5570644 28399119

[45] Tatler AL, Barnes J, Habgood A (2016). Caffeine inhibits TGF-beta activation in epithelial cells, interrupts fibroblast responses to TGF-beta, and reduces established fibrosis in ex vivo precision-cut lung slices. Thorax.

